# Supplementary material for: Polymorphisms in Pfkelch13 domains before and after the introduction of artemisinin-based combination therapy in Southwest Nigeria
Source: PLoS One. 2025 Mar 31;20(3):e0316479. doi: 10.1371/journal.pone.0316479 (PMC11957316; doi:10.1371/journal.pone.0316479)

Samples: 14485
Bases: 826
Average spacing: 18

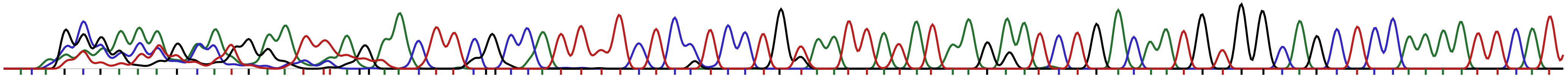

10 20 30 40 50 60 70 80

ACAGCGAAAGCATGAATTTAGGAACTTCGGCCATTTTCTCCTCCTGTAATTATATAAGAATCTGACAATGTGGCAGCTCCAAAATTCAT

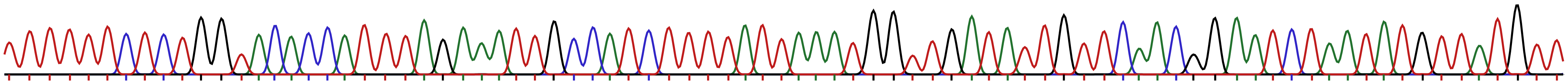

90 100 110 120 130 140 150 160 170

TTTTTTCTCTGGTACACCATTTAGAAATTGCCATCTTTTATTAAATGGTTGATATTGTTCAACGGAATCTAATATGTTATGTT

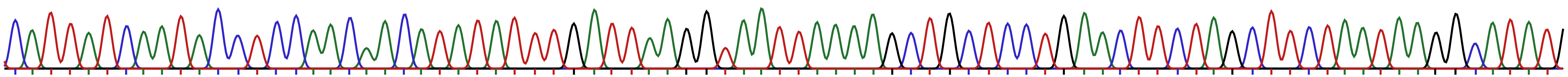

180 190 200 210 220 230 240 250

CATTATCAATACCTCCAACAACATATATTTGATTAAGGTAATTAAAAGCTGCTCCTGAACTTCTAGCTTCTAATAAGGCATAT

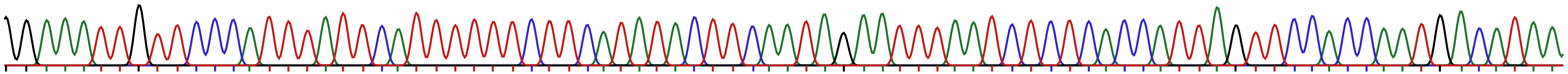

260 270 280 290 300 310 320 330

GGAAATTGTTCCCATTTATTCATTTTTTCTTCATATACTTCAATAGAATTTAATCTCTCACCATTAGTTCCACCAATGACATAA

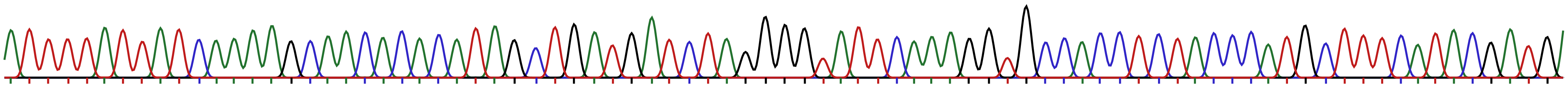

340 350 360 370 380 390 400 410 420

ATTTTATTATCAAAAGCAACACACATAGCTGATGATCTAGGGGTATTCAAAGGTGCCACCTCTACCCATGCTTTCATACGATG

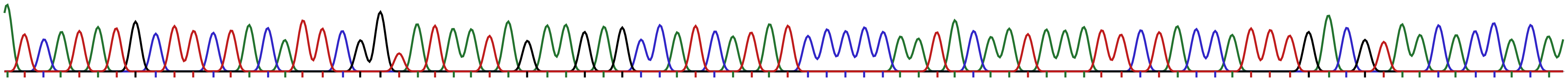

430 440 450 460 470 480 490 500

ATCATATGCTTCTACATTCGGTATAATAGAAGAGCCATCATATCCCCCAATACAATAAATTCTACCATTTGACGTAACACCACA

Samples: 14485
Bases: 826
Average spacing: 18

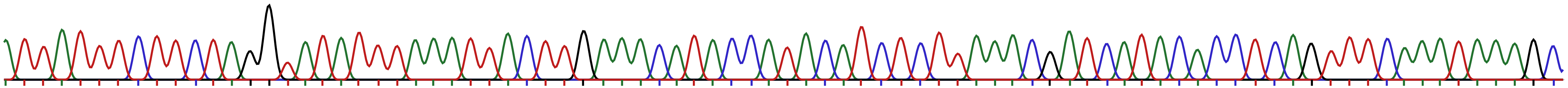

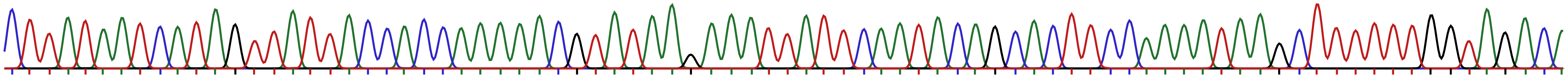

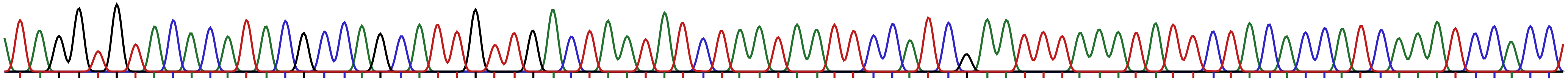

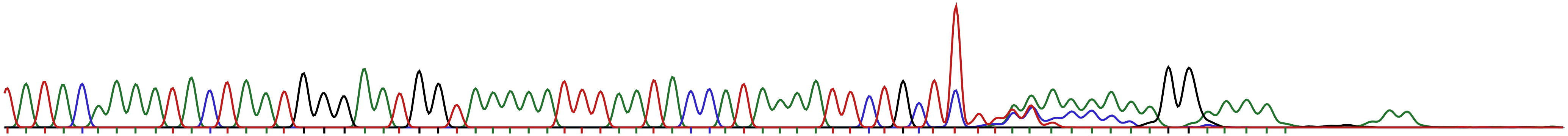

Samples: 14485
Bases: 826
Average spacing: 18

8/17/2022

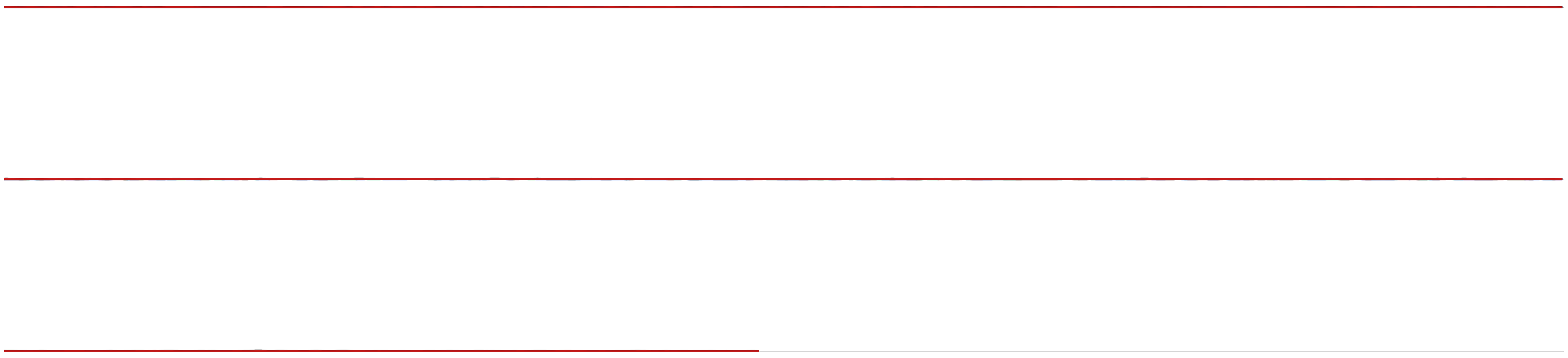

Supplement: Supporting information 2 — (ZIP) [file pone.0316479.s002.zip › 069KN1R_PREMIX_Plate_KELCH1_E04.pdf]
